# Supplementary material for: Associations Between Overparenting and Offspring’s Mental Health: A Meta-Analysis of Multiple Moderators
Source: Behav Sci (Basel). 2025 Sep 11;15(9):1235. doi: 10.3390/bs15091235 (PMC12466707; doi:10.3390/bs15091235)
Supplement: Supplementary file 1 [file behavsci-15-01235-s001.zip › behavsci-3745778-supplementary.pdf]

# Supplemental Materials

## Supplemental Tables

1. Table S1: The Q Statistics and the Omnibus Test of the Moderators between Overparenting and depression
2. Table S2: The Q Statistics and the Omnibus Test of the Moderators between Overparenting and Anxiety
3. Table S3: The Q Statistics and the Omnibus Test of the Moderators between Overparenting and Life Satisfaction
4. Table S4: The Q Statistics and the Omnibus Test of the Moderators between Overparenting and Subjective Well-being

**Table S1** The Q Statistics and the Omnibus Test of the Moderators between Overparenting and Depression

| Moderator | *k* | *n* | *r* | 95%CI | *Qb*（*df*） | *p* |
|---|---|---|---|---|---|---|
| country | 65 | 31855 | 0.185 | 0.162, 0.208 | 15.747(5) | 0.008 |
| offspring's gender | 65 | 31855 | 0.204 | 0.168, 0.240 | 0.121(5) | 0.728 |
| parental gender | 65 | 31855 | 0.179 | 0.144, 0.213 | 7.499(2) | 0.024 |
| informants | 65 | 31855 | 0.190 | 0.160, 0.219 | 1.221(1) | 0.269 |
| study design | 65 | 31855 | 0.201 | 0.164, 0.237 | 0.319(1) | 0.572 |
| culture | 65 | 31855 | 0.189 | 0.157, 0.222 | 2.747(2) | 0.253 |
| offspring's age group | 65 | 31855 | 0.138 | 0.114, 0.163 | 50.500(2) | 0.000 |

**Table S2** The Q Statistics and the Omnibus Test of the Moderators between Overparenting and Anxiety

| Moderator | *k* | *n* | *r* | 95%CI | *Qb*（*df*） | *p* |
|---|---|---|---|---|---|---|
| country | 64 | 37872 | 0.175 | 0.145, 0.204 | 24.469(4) | 0 |
| offspring's gender | 64 | 37872 | 0.159 | 0.126, 0.195 | 0.00(0) | 1.000 |
| parental gender | 64 | 37872 | 0.156 | 0.126, 0.186 | 21.148(3) | 0.000 |
| informants | 64 | 37872 | 0.151 | 0.122, 0.184 | 0.463(3) | 0.496 |
| study design | 64 | 37872 | 0.114 | 0.090, 0.138 | 10.271(1) | 0.001 |
| culture | 64 | 37872 | 0.160 | 0.125, 0.194 | 0.541(2) | 0.763 |
| offspring's age group | 64 | 37872 | 0.078 | 0.051, 0.105 | 77.347(2) | 0.000 |

**Table S3** The Q Statistics and the Omnibus Test of the Moderators between Overparenting and Life Satisfaction

| Moderator | *k* | *n* | *r* | 95%CI | *Qb*（*df*） | *p* |
|---|---|---|---|---|---|---|
| country | 36 | 17293 | 0.128 | 0.104, 0.151 | 31.580 (3) | 0.000 |
| offspring's gender | 36 | 17293 | 0.090 | 0.045, 0.134 | 0.246 (1) | 0.620 |
| parental gender | 36 | 17293 | 0.087 | 0.039, 0135 | 0.324 (1) | 0.569 |
| informants | 36 | 17293 | 0.070 | 0.025, 0.114 | 3.687 (1) | 0.055 |
| study design | 36 | 17293 | 0.077 | 0.030, 0.123 | 10.914 (1) | 0.001 |
| culture | 36 | 17293 | 0.132 | 0.107, 0.156 | 28.398 (2) | 0.000 |
| offspring's age group | 36 | 17293 | 0.086 | 0.038, 0.133 | 0.000 (0) | 1.000 |

**Table S4** The Q Statistics and the Omnibus Test of the Moderators between Overparenting and Subjective Well-being

| Moderator | *k* | *n* | *r* | 95%CI | *Qb*（*df*） | *p* |
|---|---|---|---|---|---|---|
| country | 4 | 1687 | 0.030 | -0.017, 0.078 | 13.328 (2) | 0.001 |
| offspring's gender | 4 | 1687 | -0.013 | -0.122, 0.097 | 0.000 (0) | 1.000 |
| parental gender | 4 | 1687 | 0.051 | -0.005, 0.107 | 1.118 (2) | 0.572 |
| informants | 4 | 1687 | -0.013 | -0.122, 0.097 | 0.000 (0) | 1.000 |
| study design | 4 | 1687 | -0.013 | -0.122, 0.097 | 0.000 (0) | 1.000 |
| culture | 4 | 1687 | 0.030 | -0.017, 0.078 | 13.328 (2) | 0.001 |
| offspring's age group | 4 | 1687 | 0.061 | -0.005, 0.107 | 1.088 (1) | 0.297 |

## Supplemental Figures

1. Figure S1: Funnel Plot for Overparenting and depression
2. Figure S2: Funnel Plot for Overparenting and Anxiety
3. Figure S3: Funnel Plot for Overparenting and Life Satisfaction
4. Figure S4: Funnel Plot for Overparenting and Subjective Well-being

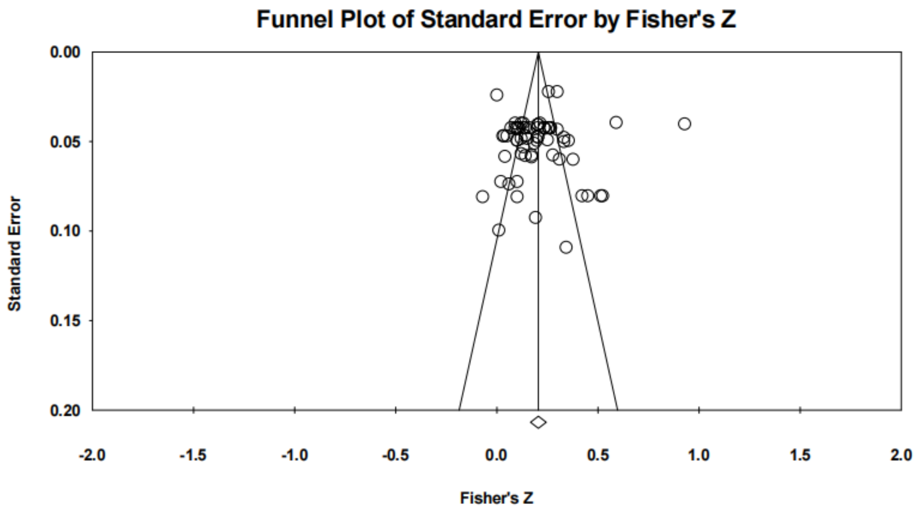


**Figure S1** *Funnel Plot for Overparenting and Depression*

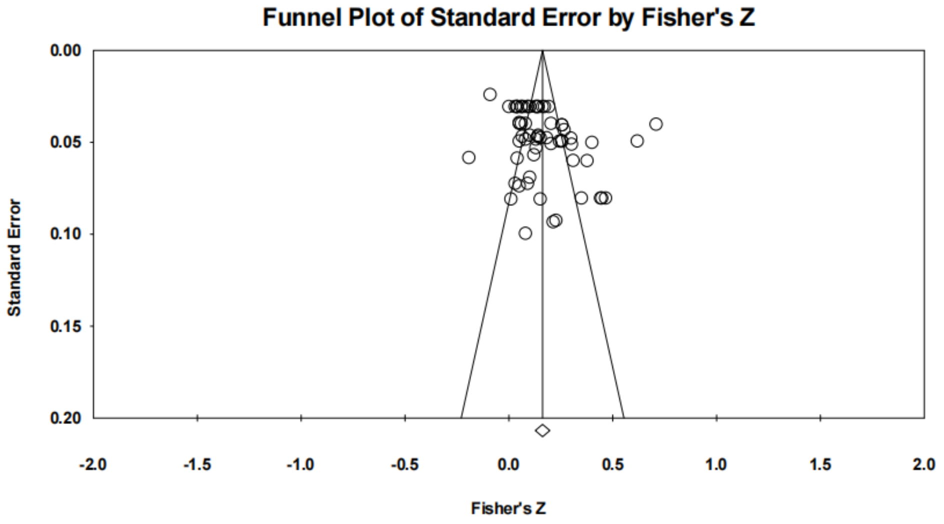


**Figure S2** *Funnel Plot for Overparenting and Anxiety*

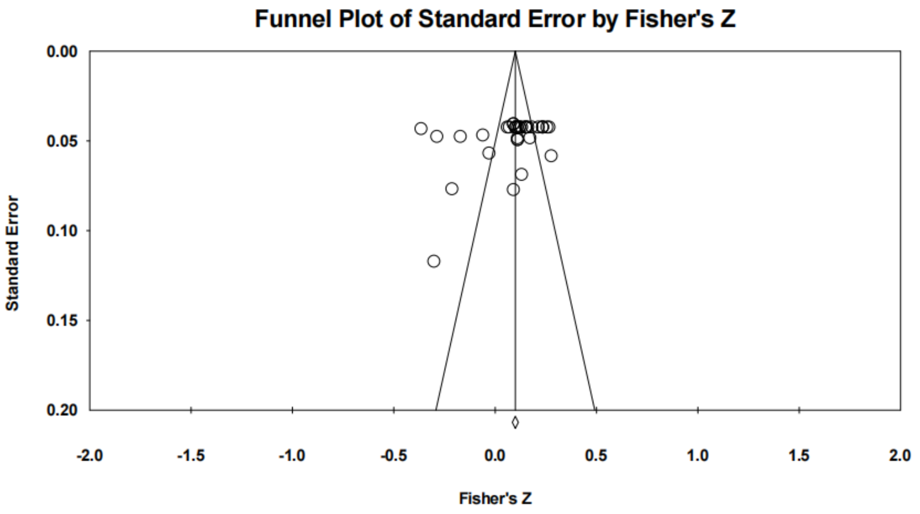


**Figure S3** *Funnel Plot for Overparenting and Life Satisfaction*

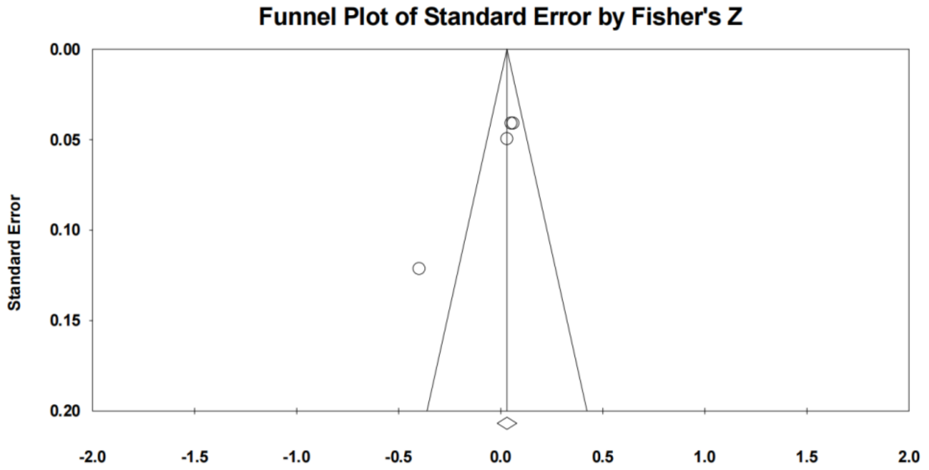


**Figure S4** *Funnel Plot for Overparenting and Subjective Well-being*
